# A Study to Assess the Pancreatitis Three-Factor Scoring System in Comparison With Computed Tomography (CT) Severity Index Scoring in Predicting the Severity of Acute Pancreatitis

**DOI:** 10.7759/cureus.89188

**Published:** 2025-08-01

**Authors:** Anirudh Arora, Arulappan T, Sivaraja PK

**Affiliations:** 1 General Surgery, Sri Ramachandra Institute of Higher Education and Research, Chennai, IND

**Keywords:** acute pancreatitis, ct severity index, diagnostic accuracy, panc3 score, risk stratification

## Abstract

Background: Acute pancreatitis is a common gastrointestinal emergency with potentially severe forms requiring early risk prediction for effective management. This study evaluated the diagnostic performance of the PANC3 scoring system in predicting severity and compared it with the CT Severity Index (CTSI).

Methods: A prospective, non-randomised study was conducted at a tertiary care centre in Chennai from January 2020 to November 2021, involving 60 patients diagnosed with acute pancreatitis within 48 hours of symptom onset. PANC3 scoring was based on haematocrit, BMI, and pleural effusion. All patients underwent contrast-enhanced CT at 48 hours to determine the CTSI. Statistical analysis included chi-square testing and the evaluation of the receiver operating characteristic (ROC) curve.

Results: Among 60 patients, 25 (41.7%) were classified as severe acute pancreatitis (SAP) by the CTSI. Of these, 10 (16.7%) had a positive PANC3 score (score = 3), all of whom (100%) had severe disease, yielding a specificity and a positive predictive value of 100% and a sensitivity of 40%. The overall diagnostic accuracy of the PANC3 score was 75% (AUC = 0.700, p = 0.009). BMI >30 kg/m² (41; 68.3%) and pleural effusion (30; 50%) were significantly associated with severity, while elevated haematocrit >44% (22; 36.6%) was not.

Conclusion: PANC3 demonstrates high specificity and positive predictive value for early identification of SAP, offering a simple and cost-effective risk stratification tool when used alongside established scoring systems.

## Introduction

Acute pancreatitis is a relatively common yet potentially life-threatening condition marked by inflammation of the pancreas, which may extend to involve surrounding tissues and can lead to multi-organ dysfunction syndrome [1]. Despite advances in supportive care, the associated mortality remains significant. The global incidence of acute pancreatitis varies, largely influenced by regional differences in causative factors, most notably, alcohol intake and the prevalence of gallstone disease. Additionally, cigarette smoking has been identified as an independent risk factor. Approximately one in four cases may progress to severe disease with complications, and the overall mortality has remained between 10% and 25% over the past two decades [2].

Acute pancreatitis is classified based on the Revised Atlanta Classification (2012) as mild, moderately severe, or severe. Mild acute pancreatitis involves interstitial oedema without organ failure or local/systemic complications and is usually self-limiting. Moderately severe cases involve transient organ failure or local complications, while severe cases are characterised by persistent organ failure, often requiring intensive care support [3]. Therefore, risk stratification tools are essential for guiding appropriate management strategies. The global incidence of acute pancreatitis has shown a rising trend over the past few decades, which is largely attributed to lifestyle-related factors such as the increasing prevalence of gallstone disease and alcohol abuse. Gallstones remain the leading cause of acute pancreatitis in many countries, particularly among older adults and women, while alcohol-related pancreatitis is more common in younger male patients. This rise may also reflect improved diagnostic imaging and greater awareness of the disease among healthcare professionals [4,5]. While most cases are mild and self-limiting, approximately 20% progress to severe acute pancreatitis (SAP), which carries a mortality rate of over 30%. These patients require critical care to manage fluids and vital signs effectively [6]. Early and accurate severity assessment is essential for guiding treatment, predicting outcomes, and optimising resource use for acute pancreatitis [7].

Scoring systems and clinical evaluations help identify patients who require intensive care for timely intervention and treatment. Although many scoring systems exist, only a few are widely used in clinical settings. These systems rely on clinical, biochemical, or radiological factors to assess severity [8]. An ideal tool should have high sensitivity and predictive value, detect necrosis early (within 48 h), be quick to perform (within 4 h), be widely available, inexpensive, and objective. Most scoring systems focus on the body's systemic response to acute pancreatitis to predict severity and mortality [9].

Ranson introduced the first numeric scoring system in 1974, based on clinical and hematochemical variables, with mortality rising as more risk factors are present [10]. The Glasgow (Imrie) criteria, developed in 1985, also predict mortality [11]. More recently, the APACHE II system has gained popularity for its reliability. It is a general ICU scoring tool that assesses overall disease severity using 12 physiological variables, including temperature, blood pressure, and arterial oxygen levels [12,13]. Although more complex and time-consuming, it allows daily reassessment, making it useful for monitoring disease progression.

For prognostication in acute pancreatitis, various biochemical markers have been investigated. Procalcitonin has shown utility particularly in predicting infected pancreatic necrosis and systemic complications, though it is not routinely recommended within 12 hours for initial severity assessment [14,15]. Additionally, hypocalcemia and elevated LDH levels are part of conventional severity scoring systems like Ranson’s criteria and have been associated with increased risk of complications and mortality in acute pancreatitis [16,17].

In 1985, Balthazar developed a CT-based scoring system to assess the severity of acute pancreatitis by evaluating local pancreatic and peripancreatic changes. Contrast-enhanced CT helps identify necrosis through reduced tissue enhancement due to impaired perfusion [18]. This study evaluated the effectiveness of the PANC3 scoring system in predicting the severity of acute pancreatitis using simple, rapidly obtainable parameters: haematocrit, BMI, and pleural effusion on chest X-ray. Compared to imaging-based tools like the CT Severity Index (CTSI), PANC3 offers a practical and cost-effective bedside method that can facilitate early triage, particularly in settings where access to cross-sectional imaging is limited. Therefore, this study aimed to compare the PANC3 and CTSI scoring systems for severity assessment in patients with acute pancreatitis in a South Indian tertiary care population.

## Materials and methods

This prospective, non-randomised study was carried out on 60 patients in the Department of General Surgery at Sri Ramachandra Institute of Higher Education and Research, Chennai, between January 2020 and November 2021. Ethical clearance was obtained from the Institutional Ethics Committee (IEC No. CSP-MED/20/JAN/58/16), and written informed consent was secured from all participants prior to their enrolment in the study.

Inclusion criteria

Patients aged over 18 years were included if they met at least two of the internationally accepted diagnostic criteria for acute pancreatitis: (i) characteristic abdominal pain (typically epigastric pain radiating to the back), (ii) serum amylase and/or lipase levels ≥3 times the upper limit of normal, and (iii) imaging findings consistent with acute pancreatitis. Additionally, only patients presenting within 48 hours of symptom onset were enrolled, to allow early assessment using the PANC3 score.

Exclusion criteria

The study excluded patients with recurrent or acute-on-chronic pancreatitis, as well as those with pre-existing cardiac, hepatic, or renal failure since these systemic comorbidities can independently influence biochemical markers (e.g., haematocrit, serum creatinine) and radiological findings, thereby confounding the assessment of severity using the PANC3 score and CTSI. Additionally, patients with such comorbidities may have a different baseline risk profile for complications, which could skew the interpretation of severity stratification. Patients presenting more than 48 hours after symptom onset were also excluded, as delayed presentation may affect the accuracy of early prognostic markers, particularly PANC3, which is designed for predicting early severity.

Methods

Patients who fulfilled the inclusion criteria were enrolled upon presentation to the surgical casualty or outpatient department, provided the onset of symptoms was within 48 hours, as determined by clinical history. This time frame was chosen to allow for early prognostic assessment. To minimise confounding variables, patients referred from other healthcare facilities were excluded if prior administration of intravenous fluids, analgesics, or imaging could not be reliably documented, or if presentation occurred beyond 48 hours from symptom onset.

At the time of hospital admission, before any resuscitative intervention or analgesic administration, baseline parameters were collected. Body mass index (BMI) was calculated using measured height and weight, haematocrit was measured from a venous blood sample, and an upright chest radiograph (CXR) was obtained to assess for the presence of pleural effusion. These values were used to calculate the PANC3 score.

The PANC3 scoring system, originally proposed by Brown et al. [19], comprises three parameters: haematocrit greater than 44%, BMI exceeding 30 kg/m², and the presence of pleural effusion on chest X-ray. One point is assigned for each positive parameter, resulting in a cumulative score ranging from 0 to 3. By previously validated studies, a total score of 3 was defined as PANC3-positive, predictive of SAP, while scores of 0 to 2 were considered PANC3-negative. Although intermediate scores of 1 or 2 may represent varying degrees of risk, this dichotomisation was adopted to align with existing literature and to facilitate binary statistical analysis, including receiver operating characteristic (ROC) evaluation.

The severity of acute pancreatitis for all enrolled patients was determined using the Revised Atlanta Classification (2012) [3], applied after evaluation with CECT at 48 hours after symptom onset. This classification categorises disease severity into mild, moderately severe, and severe acute pancreatitis based on the presence and duration of organ failure and the occurrence of local or systemic complications. The CTSI calculated from CECT findings was used to quantify the extent of pancreatic and peripancreatic inflammation and necrosis.

The diagnostic performance metrics presented were calculated using predefined cutoff values derived from the original PANC3 scoring system proposed by Brown et al. [19]. The cutoff values applied were haematocrit >44%, BMI >30 kg/m², and presence of pleural effusion on chest X-ray (binary classification: present/absent). Each parameter was evaluated individually against the reference standard of SAP as defined by the CTSI. These cutoffs were consistently applied throughout the analysis to ensure reproducibility of sensitivity, specificity, positive predictive value (PPV), and negative predictive value (NPV) calculations.

A CECT scan of the abdomen using a pancreatic protocol was scheduled 48 hours after the onset of abdominal pain, following radiological guidelines. This timing was selected to ensure optimal visualisation of pancreatic necrosis and peripancreatic inflammatory changes and to allow for accurate calculation of the CTSI based on the Balthazar grading system.

Statistical analysis

Data were summarised using mean, standard deviation, frequencies, and percentages. Continuous variables were analysed with the independent t-test, while categorical variables were compared using Pearson’s chi-square test. ROC analysis was used to determine cutoff values, with cross-tabulation employed to assess sensitivity and specificity. A p-value < 0.05 (two-tailed) was considered statistically significant. Analysis was conducted using IBM SPSS Statistics for Windows, Version 21 (Released 2012; IBM Corp., Armonk, New York, United States).

## Results

Of the 60 patients with acute pancreatitis, the mean age was 43.5 ± 8 years, and the median age was 41.5 years. The majority of patients (16; 26.7%) belonged to the 31-40 years age group. The number of male patients was 47 (78.3%). Elevated haematocrit levels >44% were observed in 22 patients (36.6%), BMI >30 kg/m² in 41 patients (68.3%), and pleural effusion on chest radiography in 30 patients (50%). Based on the PANC3 score, 10 (16.7%) tested positive (score = 3), indicating potential severe pancreatitis. According to the CTSI, two (3.3%) patients had mild pancreatitis, 33 (55.0%) had moderate pancreatitis, and 25 (41.7%) had severe pancreatitis (Table 1).

**Table 1:** Demographic profile, PANC3 parameters, and CTSI classification BMI: Body Mass Index; CTSI: CT Severity Index

Parameter	Category	N(%)
Age group (years)	18–30	13(21.7%)
	31–40	16(26.7%)
	41–50	13(21.7%)
	51–60	6(10%)
	61–70	8(13.3%)
	>70	4(6.7%)
Gender	Male	47(78.3%)
	Female	13(21.7%)
Haematocrit (%)	≤ 44	38(63.3%)
	> 44	22(36.6%)
BMI (kg/m²)	≤ 30	19(31.7%)
	> 30	41(68.3%)
Pleural Effusion (Chest X-ray)	Present	30(50%)
	Absent	30(50%)
PANC3 Score	Positive (score = 3)	10(16.7%)
	Negative (score < 3)	50(83.3%)
CTSI Severity Classification	Mild	2(3.3%)
	Moderate	33(55%)
	Severe	25(41.7%)

Of the 60 patients enrolled in the study, 10 patients (16.7%) were classified as severe by PANC3 scoring (score = 3), while 25 patients (41.7%) were classified as severe based on the CTSI. This discrepancy reflects a lower sensitivity but higher specificity of the PANC3 score in identifying SAP compared to the CTSI.

To evaluate the diagnostic performance of the PANC3 score, sensitivity, specificity, PPV, and NPV were calculated using the CTSI as the reference standard. The sensitivity of the PANC3 score was 40.0% (95% CI: 21.1%-61.3%), and specificity was 100.0% (95% CI: 92.9%-100%). The PPV was 100.0% (95% CI: 69.1%-100%), and the NPV was 70.0% (95% CI: 56.4%-81.0%).

Of the 60 patients, 10 (16.7%) had a positive PANC3 score, meaning all three parameters: haematocrit >44%, BMI >30 kg/m², and pleural effusion on chest X-ray were present. The remaining 50 patients (83.3%) had a negative PANC3 score, with varying combinations of absent parameters. Among these, 28 patients had only one positive parameter, while 22 had two positive parameters but did not meet all three criteria. The most frequently absent parameter was pleural effusion on chest X-ray, which was missing in 38 of the 50 PANC3-negative cases (76%). Haematocrit ≤44% was absent in 25 patients (50%), and BMI ≤30 kg/m² was absent in 21 patients (42%).

Among the 60 patients, 25 (41.7%) were classified as having SAP based on the CTSI. Of these 25 severe cases, only 10 (40%) had a positive PANC3 score, while the remaining 15 (60%) were PANC3 negative, indicating a substantial false-negative rate. This reflects a sensitivity of only 40%, suggesting that the PANC3 score failed to identify the majority of radiologically severe cases. However, the PANC3 score demonstrated excellent specificity. None of the patients with mild (n = 2) or moderate (n = 33) pancreatitis based on CTSI were PANC3 positive; all 35 were PANC3 negative, yielding a specificity of 100%. Thus, while a positive PANC3 score was strongly predictive of severe disease (PPV 100%), its limited sensitivity (40%) restricts its ability to serve as a standalone screening tool for severity stratification.

All 60 patients underwent follow-up with CECT performed at 48 hours after symptom onset, which was used to calculate CTSI. The severity classification into mild, moderate, and SAP was based on the Revised Atlanta Classification (2012), which considers both local complications and the presence or persistence of organ failure. Radiological features of necrosis and peripancreatic inflammation, as visualised on CECT, informed the CTSI scores used for categorisation.

A significant association was observed between PANC3 positivity and SAP based on CTSI classification (χ² = 16.800, p = 0.0005). However, this p-value denotes a robust statistical relationship. The sensitivity of the PANC3 score was only 40.0%, indicating that this scoring system did not identify 60% of patients with severe disease. This limits its utility as a standalone screening tool for early severity prediction. In contrast, the specificity of the PANC3 score was 100.0%, as no patients with mild or moderate pancreatitis were PANC3-positive. The PPV was also 100.0%, while the NPV was 70.0%. The AUC was 0.70, which shows that it may not detect all severe cases (Tables 2, 3).

**Table 2:** Comparison of severity of acute pancreatitis (CT severity index) with PANC3 CTSI: CT Severity Index

Severity of Acute Pancreatitis (CTSI)	Category	PANC3		p-value
		Positive	Negative	
Non-severe	Mild	0	2(4%)	0.0005
	Moderate	0	33(66%)	
Severe	Severe	10(100%)	15(30%)	

**Table 3:** ROC curve for PANC3 in predicting SAP SAP: Severe Acute Pancreatitis; ROC: Receiver Operating Characteristic; PPV: Positive Predictive Value; NPV: Negative Predictive Value

Test	PANC3
Sensitivity	40%
Specificity	100%
PPV	100%
NPV	70%
Accuracy	75%

A comparison of PANC3 and the CTSI showed that haematocrit levels were not significantly associated with disease severity. Although a higher proportion of patients with haematocrit >44% had severe pancreatitis (59.1%) than those with haematocrit ≤44% (31.6%), the difference did not reach significance (χ² = 4.878, p = 0.087). In contrast, BMI was significantly associated with disease severity. Among patients with BMI >30, 73.7% had severe disease, compared to only 26.8% of those with BMI ≤30 (χ² = 12.977, p = 0.0005). Similarly, the presence of pleural effusion on chest radiography was strongly associated with severe pancreatitis. A total of 63.3% of patients with pleural effusion had severe pancreatitis, while only 20% of those without pleural effusion had severe pancreatitis (χ² = 12.427, p = 0.002) (Table 4).

**Table 4:** Association of haematocrit, BMI, and pleural effusion with severity of acute pancreatitis (CTSI Classification) BMI: Body Mass Index; CTSI: CT Severity Index

Parameter	Category	CTSI, N(%)			p-value
		Mild	Moderate	Severe	
Haematocrit (%)	≤ 44	1(2.6%)	25(65.8%)	12(31.6%)	0.087
	> 44	1(4.5%)	8(36.4%)	13(59.1%)	
BMI (kg/m²)	≤ 30	1(2.4%)	29(70.7%)	11(26.8%)	0.0005
	> 30	1(5.3%)	4(21.1%)	14(73.7%)	
Pleural Effusion	Present	0	11(36.7%)	19(63.3%)	0.002
	Absent	2(6.7%)	22(73.3%)	6(20%)	

Haematocrit showed moderate diagnostic performance with an AUC of 0.631 (95% CI: 0.478-0.768), sensitivity of 52.0%, specificity of 74.3%, PPV of 59.1%, NPV of 68.4%, and an overall accuracy of 65.0%; however, this was not statistically significant (p = 0.085). In contrast, BMI demonstrated significant predictive value, with an AUC of 0.709 (95% CI: 0.565-0.830; p = 0.006), sensitivity of 56.0%, specificity of 85.7%, PPV of 73.7%, NPV of 73.2%, and accuracy of 73.3%. Pleural effusion exhibited the strongest diagnostic association, yielding an AUC of 0.737 (95% CI: 0.593-0.852; p = 0.002), with sensitivity of 76.0%, specificity of 71.4%, PPV of 65.5%, NPV of 80.6%, and accuracy of 73.3% (Table 5).

**Table 5:** ROC analysis of predictors for severity of acute pancreatitis AUC: Area under the Curve; PPV: Positive Predictive Value; NPV: Negative Predictive Value; ROC: Receiver Operating Characteristic

Parameter	AUC (95% CI)	p-value	Sensitivity (%)	Specificity (%)	PPV (%)	NPV (%)	Accuracy (%)
Haematocrit >44%	0.631 (0.478–0.768)	0.085	52	74.3	59.1	68.4	65
BMI >30 kg/m²	0.709 (0.565–0.830)	0.006	56	85.7	73.7	73.2	73.3
Pleural Effusion	0.737 (0.593–0.852)	0.002	76	71.4	65.5	80.6	73.3

## Discussion

In this study, we compared the efficacy of the PANC3 scoring system in predicting the severity of acute pancreatitis with that of the CTSI. PANC3 is effective for the early diagnosis and triage of SAP, with BMI and pleural effusion showing significant values. Therefore, the distribution of non-severe disease was 58.3%, and that of SAP was 41.7%. Comparison of acute pancreatitis severity based on the CTSI with PANC3 showed a significant association (p = 0.0005). Similar results were reported in the study by Meena et al. comparing PANC 3 with Modified Atlanta criteria, APACHE II scoring, CTSI score, and CRP with p = 0.005 [20].

Our study findings showed that the PANC3 scoring system demonstrated a sensitivity of 40%, specificity of 100%, PPV of 100%, NPV of 70%, and overall accuracy of 75%. The association between PANC3 positivity and SAP (as defined by the CTSI) was highly significant (p = 0.009). This result was confirmed by the study by Beduschi et al., who reported a sensitivity of 50%, specificity of 100%, PPV of 100%, and NPV of 90.6%, and also found a significant association (p < 0.01). Their findings reinforce the high specificity and PPV of the PANC3 score, consistent with our results [21]. Similarly, Meena et al. observed a sensitivity of 81.82%, specificity of 92.31%, PPV of 75%, and NPV of 94.7%, with a significant p = 0.005. However, their reported sensitivity was higher [20]. This is similar to our study, which confirms the strong predictive value of a positive PANC3 score for SAP (p = 0.009).

Fukuda et al. also found high specificity (100%) and PPV (81.66%), although with lower sensitivity (31.25%) and NPV (83.07%). These results align with our findings, further supporting the reliability of PANC3 in confirming severe cases when positive [22]. Hence, in the study by Lankisch et al., the value of haemoconcentration lies in its high NPV; this supports our study findings, with an NPV of 68.4% [23]. However, the sensitivity of the PANC3 score was notably limited at 40%, and the AUC was only 0.70, indicating moderate overall diagnostic performance. These findings underscore the restricted ability of the score to detect all severe cases.

In our study, haematocrit as a predictor of SAP demonstrated a sensitivity of 52%, specificity of 74.3%, PPV of 59.1%, and NPV of 68.4%, with no significant association (p = 0.085) between elevated haematocrit and severe disease as classified by the CTSI. These findings suggest that while the haematocrit level shows moderate specificity, its overall predictive value for SAP is limited. This result aligns with the study by Remes-Troche et al., which found a sensitivity of 59%, specificity of 35%, PPV of 21%, and NPV of 74% (p = 0.23), indicating that haematocrit as a single parameter lacks sufficient accuracy to reliably predict SAP [24]. In our study, haematocrit >44% did not show a significant association with severe pancreatitis, suggesting that its contribution to the overall score may warrant reconsideration in certain clinical populations.

In our study, pleural effusion on chest X-ray emerged as a significant early predictor of SAP, with a sensitivity of 76%, specificity of 71.4%, PPV of 65.5%, and NPV of 80.6% (p = 0.002), in comparison with the CT Severity Index. These findings align with those of Brown et al., who reported higher sensitivity (84%), specificity (91%), PPV (62%), and NPV (97%), emphasising the strong presence of pleural effusion. Together, these results support the utility of chest X-ray-detected pleural effusion as a valuable early marker for risk stratification in acute pancreatitis, particularly for ruling out severe disease [19].

In our study, BMI was found to be a significant predictor of SAP, with a sensitivity (56%), specificity (85.7%), PPV (73.7%), NPV (73.2%), and a significant association (p = 0.006) with severe disease. This finding aligns with a study by Türkoğlu et al., who also found BMI to be significantly higher in patients with severe pancreatitis (p < 0.0001) [25]. The sensitivity observed in our study was lower than that previously reported in the original study by Brown et al. (2007), which may reflect differences in sample characteristics, BMI distribution, or timing of imaging. Such inter-study variability highlights the need for further external validation. Given the stronger predictive value of BMI >30 kg/m² and pleural effusion, the utility of a simplified two-variable model excluding haematocrit should be explored in future studies to improve sensitivity without substantially compromising specificity [19]. 

This study highlights the high specificity and predictive value of the PANC3 score, especially its components, BMI and pleural effusion, in the early identification of SAP. Its simplicity and cost-effectiveness make it a useful tool for triage in resource-limited settings. In situations where contrast-enhanced CT is not immediately available, the PANC3 score can facilitate early clinical decision-making and referral by identifying patients at risk of severe disease based on readily available parameters. Additionally, this study did not evaluate alternative cutoff thresholds for the individual components of the PANC3 score. Exploration of such thresholds could help optimise the trade-off between sensitivity and specificity in different clinical settings. Future research should validate these findings in larger multicentre cohorts and assess the utility of PANC3 across diverse populations and clinical settings.

Limitations

This study is limited by its single-centre design, modest sample size, and lack of long-term follow-up. A key limitation is the low sensitivity (40%) of the PANC3 score, which restricts its ability to identify all severe cases of acute pancreatitis and makes it unsuitable for use as a standalone screening tool. Additionally, haematocrit >44% did not show a significant association with disease severity in this study, potentially weakening the reliability of the full three-parameter model. This raises the need to re-evaluate the scoring system and consider simplified variants. The exclusion of patients with significant comorbidities also limits the generalizability of the findings to broader clinical populations.

## Conclusions

The PANC3 score, due to its high specificity and PPV, demonstrates potential as a rule-in tool for SAP in early clinical triage, particularly in resource-limited settings. However, its low sensitivity limits its ability to reliably exclude severe disease, and therefore it should not be used as a standalone screening tool for early identification of all SAP cases. Furthermore, the lack of a significant association between haematocrit and severity in this cohort raises concerns about the robustness of the full three-parameter model. These findings support the need for further investigation into simplified or revised versions of the PANC3 score, possibly focusing on parameters such as BMI and pleural effusion, which showed a stronger predictive value.
